# Health disparities and socioeconomic challenges faced by the Khawaja Sira (intersex and gender-diverse) community in Islamabad, Pakistan: A descriptive cross-sectional study

**DOI:** 10.1371/journal.pgph.0005798

**Published:** 2026-07-10

**Authors:** Enishwa Ali, Mariam Ashraf

**Affiliations:** 1 Department of Public Health, Bahria University, Islamabad, Pakistan; 2 School of Public Health, Health Services Academy, Islamabad, Pakistan; Aga Khan University, PAKISTAN

## Abstract

In Pakistan, gender-diverse and intersex individuals locally identified as Khawaja Sira face systemic exclusion, social stigma, and limited access to essential health services. Despite the 2018 Transgender Persons (Protection of Rights) Act, empirical evidence on their health conditions and socio-economic realities remains limited. This study assesses the physical and mental health status, healthcare access, and socio-economic challenges of the Khawaja Sira community in Islamabad. A descriptive cross-sectional study recruited 91 self-identified Khawaja Sira individuals in Islamabad through snowball sampling initiated via community leaders (“Gurus”). Data were collected using structured questionnaires incorporating the Hopkins Symptom Checklist (HSCL-25; Cronbach’s α = 0.92) for mental health and the Health Protective Behaviour Scale (HPBS; α = 0.89). Descriptive statistics were analyzed using SPSS Version 19. Recruitment through community gatekeepers likely introduced network bias, potentially limiting representativeness. Within the sample, the mean HSCL-25 score was 2.1 (SD = 0.6), with 68% of participants exceeding the clinical cut-off of 1.75 for significant psychological distress. Common physical conditions included hypertension (20%), diabetes (9%), and asthma (7%). Only 25% reported regular health check-ups, and 73% lacked awareness of blood sugar levels. Tobacco use was reported by 57% of participants. Unemployment affected 34% of the sample, and 66% reported insufficient income for basic expenses. Nearly half (45%) felt uncomfortable or unsafe in healthcare settings, citing stigma and discrimination. This exploratory study documents substantial health inequities and socio-economic challenges among Khawaja Sira individuals recruited in Islamabad, with findings that warrant further investigation in more representative samples. Findings suggest urgent need for culturally competent healthcare services, mental health support, and inclusive social protection policies.

## Background

Khwaja Sira is the most commonly used term to identify third-gender people in Pakistan, an umbrella term used in South Asia for people who were assigned male sex at birth but now identify as neither male nor female, instead recognising themselves as a third gender [1].

Currently, “transgender” describes people whose gender identity differs from the sex they were assigned at birth. This diverse group includes transmen, transwomen, and intersex individuals, whose experiences are shaped by a complex mix of societal, legal, and historical factors. Throughout history, transgender individuals in the subcontinent have played various social roles. In some cultures, they have been valued, recognised for their unique contributions and considered to hold spiritual significance. This perspective influences societal attitudes and the roles that transgender individuals fulfil. However, treatment varies across cultures; some people use derogatory language, reinforcing negative stereotypes and marginalising these individuals, while others offer them special privileges, such as ceremonial positions or community recognition. In countries like India and Pakistan, terms such as “Khawaja sira,” “Khusray,” and “Hijray” are embedded in the cultural lexicon [2].

Pakistan has advanced notably in recognizing and safeguarding transgender rights, especially through the 2018 Transgender Persons and gender diverse individuals (Protection of Rights) Act [3]. This legislation bars discrimination in education, healthcare, and employment, and empowers transgender people to update their official documents to match their self-identified gender. Pakistan also upholds international treaties, such as the UDHR and ICCPR, which emphasize dignity, security, and equality for everyone. Nevertheless, transgender individuals still encounter societal issues such as family rejection, economic struggles, and social exclusion. Many are pushed into begging or sex work due to limited opportunities. The disparity between legal protections and societal realities underscores the urgent need for increased acceptance, economic opportunities, and supportive resources for the transgender community [3]. According to the 2017 census of the Pakistan Bureau of Statistics, there are 6,709 transgenders in Punjab, 2,527 in Sindh, 913 in Khyber Pakhtunkhwa, 133 in Islamabad, and 109 in Balochistan [3].

Unfortunately, transgender people have been disregarded for a very long time in Pakistan because to their general rejection, societal stigma, and status of being “trans”. These transgender people have no place in either a public line or a place of learning where there is a distinction between men and women. Lack of access to a basic education and decent employment options causes people to obtain covert identity, fictitious names, and odd living arrangements. (The most resistant groups to accept these so-called incomplete entities are frequently their close family members who force them to migrate away from their houses and join their fellows in beginning to live in slums, ghettos, and other remote locations from mainstream society [4].

In Pakistan, the journey of the transgender community toward equality and inclusion has been an uphill battle, one that many have faced alone. For countless transgender individuals, discrimination begins at birth and often starts within their own homes. Family estrangement locks them into a lifetime of socio-economic hardship, leaving little access to the basic human rights of education, healthcare, and dignity. Despite Pakistan’s constitutional guarantee of fundamental rights for every citizen, transgender people frequently find themselves on the fringes [5].

Ten years ago, Pakistan’s census officially recognized transgender people for the first time. Only 21,774 individuals were listed – a number many within the community argue is a gross underestimation. Transaction Pakistan and other organizations speculate there may be many more, though the results of the latest 2023 national census have yet to be fully released. Such underreporting relegates transgender individuals to social invisibility, perpetuating a cycle of marginalization. Alarmingly, 92% of transgender people report experiencing violence or abuse, over 80% report being denied employment opportunities due to their gender identity and only 7% are employed in formal sectors [6]. In the political sphere, their representation is nearly non-existent, with significant barriers to voting and contesting in elections. These challenges paint a bleak picture of the systemic discrimination faced by this community and highlight the urgent need for targeted interventions [7].

In Pakistan, where cultural and religious norms often conflict with gender diversity, intersex people experience significant health disparities. This study aims to assess the health conditions, healthcare accessibility, and socio-economic issues impacting the intersex population in Islamabad, providing insights for improving healthcare policies and promoting the rights of intersex individuals.

## Defining identity and context: The Khawaja Sira community

Accurate and culturally relevant terminology is crucial for meaningful research on sexual and gender diversity, especially in South Asia. The initial conflation of intersex and transgender identities in the study was subject to strong critique, emphasizing the need for clear definitions within the Pakistani context.

### Distinguishing core identities

**Khawaja Sira:** This is the local, traditional, and culturally relevant umbrella term used in Pakistan to describe a recognised third-gender identity. *The Khawaja Sira* community generally includes individuals who identify as third gender, those who are transgender, and sometimes those with intersex variations, functioning within a specific kinship system that has deep historical roots in the subcontinent [8]. Although the Pakistani government, through the 2018 Transgender Persons (Protection of Rights) Act, has granted legal recognition to transgender individuals, the use of broad Western terminology often neglects the nuanced, deeply rooted social roles and historical marginalisation linked to the *Khawaja Sira* identity.

**Intersex** refers to individuals born with variations in sex characteristics, including chromosomal patterns, gonads, endocrine systems, or genitals, that do not fit the typical binary definitions of male or female. These variations, or Differences in Sex Development (DSD), are fundamentally biological [9].

**Transgender** describes individuals whose gender identity differs from the sex they were assigned at birth. Transgender identity is centred on psychological identity and personal experience, a distinction that must be maintained from intersex variations [10].

## Materials and methods

### Ethics statement

The Institutional Review Board, Health Services Academy, approved the research No. 7–82/IERC-HSA/2022-16 The study follows the principles outlined in the Declaration of Helsinki. All participants were informed of the research aims and objectives. Informed consent was sought from all the participants. This was done with verbal consent, and where possible, written consent was sought to ensure that all participants fully understood that the data collection was only for research purposes.

The security measures for the collected information and protocols to preserve anonymity and confidentiality were observed. No names or identifiable details were included, and the participants were deidentified in the final dataset to protect their anonymity. All participants were informed that they could refuse or withdraw from further participation at any time, without the need to provide a reason.

### Sample size and population estimates

The sample size calculation was based on the 2017 Pakistan Population Census figure of 133 transgender individuals in Islamabad [11]. However, we acknowledge this represents a significant underestimation. Community-based organizations (CBOs) in Islamabad and Rawalpindi report registration numbers exceeding 13,000 transgender women. Using the census figure to calculate our target sample (n = 99, based on 5% margin of error and 95% confidence level using RaoSoft calculator) compromises representativeness and limits generalizability.

We recognize that consultation with local CBOs and NGOs would have provided more realistic population estimates and enabled more representative sampling. This limitation affects the external validity of our findings.

### Recruitment strategy

We recruited 91 participants through snowball sampling initiated via community leaders (“Gurus”). While this approach facilitated trust and access to a hidden population (response rate: 91.9%), it introduces substantial selection bias. Participants recruited through Gurus likely belong to more connected networks, potentially excluding isolated individuals or those unaffiliated with any Guru structure.

**Methodological transparency:** Snowball sampling does not minimize selection bias but rather manages access barriers to a stigmatized population. To reduce clustering effects, we:

- Limited referrals to maximum 3 per participant
- Recruited from 5 different Guru networks across Islamabad
- Conducted interviews in private settings in Urdu to reduce social desirability bias
- Trained interviewers in culturally sensitive communication

**Alternative approaches considered:** While targeted venue-based or time-location sampling could have diversified recruitment, the absence of consistent gathering venues and security concerns limited feasibility.

### Survey instrument

We employed two validated instruments:

1. **Hopkins Symptom Checklist (HSCL-25):** 25-item scale measuring anxiety (10 items) and depression (15 items) with 4-point response scale (1 = “not at all” to 4=”extremely”). Internal consistency in our sample: overall α = 0.92; anxiety α = 0.83; depression α = 0.88. [12,13]
2. **Health Protective Behaviour Scale (HPBS):** 32-item scale across five dimensions (interpersonal support, general behaviour, self-knowledge, nutrition, healthcare) with 5-point response scale. Internal consistency: α = 0.89. [14]

**Translation process:** Instruments underwent forward translation (English to Urdu) by an independent bilingual translator, followed by back-translation by a second translator. Discrepancies were resolved through consensus.

**Pilot testing:** Questionnaires were pilot-tested with 10 participants (excluded from final sample) from similar community backgrounds. Based on feedback:

- Simplified 3 items with complex medical terminology
- Added visual aids for health awareness questions
- Confirmed comprehension for participants with low literacy

**Cultural adaptation limitations:** While translation ensured linguistic accuracy, we acknowledge limited cultural validation. Constructs like “interpersonal support” may not fully capture the lived realities of Khawaja Sira communities. Future research should employ cognitive interviewing and community validation.

**Administration:** Trained interviewers administered questionnaires verbally in Urdu to accommodate varying literacy levels. Interviews lasted 45–60 minutes and were conducted in private community spaces identified by participants.

### Inclusion and exclusion criteria

- All those who identified themselves as Khawaja Sira, transgender, aged 18 years and above, and consented to participate in the study were included in this research.

### Data management, collection and analysis

Five trained field researchers with prior experience in community-based research conducted interviews between January-March 2024. A quality assurance officer reviewed 10% of completed questionnaires daily for consistency and completeness.

We performed the following quality checks:

- Range checks for all numeric variables
- Logical consistency checks (e.g., employment status vs. income)
- Duplicate entry identification
- Missing data assessment (all < 5% per variable)

Descriptive statistics, including means, frequencies, and percentages, were computed using SPSS version 19. Basic bivariate analysis was conducted to explore associations between key variables; Chi-square tests (or Fisher’s Exact tests where cell counts were <5 were used for categorical variables (e.g., income adequacy vs regular health check-ups, employment status vs. HSCL-25 distress threshold); and Mann-Whitney U tests were used for continuous outcomes across two group comparisons. Results of these bivariate analyses are reported alongside descriptive statistics. Given the small, non-probabilistic sample, these analyses are intended to be hypothesis-generating rather than confirmatory and should be interpreted with caution. Missing data were minimal and were handled through case-wise deletion. No imputation or sensitivity analyses were performed. No inferential statistics were performed due to the exploratory nature of the study. Subgroup comparisons (e.g., by income, education, and gender identity) were examined descriptively but not tested for statistical significance. Missing data were minimal and were handled through case-wise deletion. No imputation or sensitivity analyses were performed. As this was a descriptive study with a small non-probabilistic sample, confidence intervals and multivariate adjustments were not calculated. However, the association between socio-economic factors (such as income and education) and health status was described only descriptively, suggesting potential confounding.

Out of 99 approached individuals, 91 agreed to participate (response rate: 91.9%). Eight declined, citing discomfort with sharing personal information.

All items in the survey were mandatory, resulting in little missing data. For any item left blank, the corresponding case was excluded from analysis for that specific variable only. The highest missing response rate was less than 5%.

## Results

### Sociodemographic characteristics

The study involved 91 self-identified Khawaja Sira individuals recruited in Islamabad. Of 91 participants, 72 (79%) self-identified as intersex, 10 (11%) as female, 6 (7%) as male, and 3 (3%) as transgender (Table 1)

**Table 1:** Socio-demographic charcteristics.

Category	Options	Frequency	Percent
Age	18-20	8	8.8%
	20-25	29	31.9%
	25-30	18	19.8%
	30+	36	39.6%
Gender	Male	6	6.6%
	Female	10	11.0%
	Transgender	3	3.3%
	Intersex/Khusra*	72	79.1%
Education	Primary	17	18.7%
	Secondary	3	3.3%
	Intermediate	6	6.6%
	Bachelors	15	16.5%
	Masters	4	4.4%
	Never been to school	46	50.5%
Ethnicity	Punjabi	67	73.6%
	Sindhi	7	7.7%
	Pakhtoon	10	11.0%
	Balochi	4	4.4%
	Afghani	3	3.3%
Employment	Student	15	16.5%
	Student and Employed	4	4.4%
	Full time	11	12.1%
	Part time	30	33.0%
	Unemployed	31	34.1%
Monthly Income	less than 25000	25	27.5%
	25000-30000	17	18.7%
	30000-50000	18	19.8%
	50000-75000	7	7.7%
	75000+	24	26.4%
Health Conditions	None	32	35.2%
	Blood pressure	18	19.8%
	Diabetes	8	8.8%
	Asthma	6	6.6%
Healthcare Access	Once a year	39	42.9%
	Once in six months	19	20.9%
	Once in three months	17	18.7%
	Once in a month	16	17.6%
Comfort in Healthcare	Yes	16	17.6%
	No	26	28.6%
	Sometimes	40	44.0%
	Never	9	9.9%
Substance Use	Tobacco	52	57.1%
	Alcohol	3	3.3%
	Heroin	2	2.2%
	Weed	6	6.6%

*“Intersex/ Khusra”: The high proportion of participants (79.1%) selecting this category most likely reflects cultural self-identification with a recognised third-gender status (“Khusra”) rather than biological Differences in Sex Development (DSD). This label should not be interpreted as indicating a clinical intersex condition. See Results section for full clarification.

Our study focused on individuals within the Khawaja Sira community network, which encompasses diverse gender identities beyond intersex. The term “Khawaja Sira” is a culturally specific umbrella category in Pakistan that includes third-gender individuals, transgender persons, and some with intersex variations. It is important to note that the high proportion of participants (79.1%) selecting the response option translated as “intersex” most likely reflects cultural self-identification with a socially recognised third-gender status—and possibly the Urdu equivalent “Khusra” which carries specific religious and legal legitimacy in Islamic jurisprudence—rather than indicating biological Differences in Sex Development (DSD), which occur in approximately 1.7% of the global population. The survey response option was derived from the 2018 Transgender Persons (Protection of Rights) Act’s recognized categories and was culturally validated with community representatives during piloting. Authors acknowledge this as a key limitation: the findings pertain to gender-diverse individuals within the Khawaja Sira social framework and should not be interpreted as data on biological intersex conditions. Future research should use clearer Urdu-language distinctions between cultural gender identities and biological sex variations.

**Age distribution:** Mean age was 30 years (SD = 5.2, range: 18–42). The narrow range (20–35 years for 80% of sample) suggests potential underrepresentation of older community members, possibly due to survival bias, social exclusion, or reluctance to participate.

**Educational attainment:** Half of the participants (51%, n = 46) never attended school. Educational levels varied widely: primary education (19%, n = 17), bachelor’s degrees (16%, n = 15), college attendance (7%, n = 6), and master’s degrees (4%, n = 4).

**Employment and income:** Unemployment affected 34% (n = 31), with 33% (n = 30) in part-time work, 16% (n = 15) students, 12% (n = 11) employed full-time, and 4% (n = 4) both students and employed. Income distribution showed 28% (n = 25) earning <25,000 PKR monthly, 19% (n = 18) earning 30,000–50,000 PKR, and 26% (n = 24) earning >75,000 PKR.

**Ethnic composition:** The sample comprised predominantly Punjabi participants (74%, n = 67), with representation from Pashtun (11%, n = 10), Sindhi (8%, n = 7), Balochi (4%, n = 4), and Afghan (3%, n = 3) communities. This distribution likely reflects network-based recruitment bias rather than true population proportions.

### Health status and healthcare access

**Physical health conditions:** Thirty-five percent (n = 32) reported no diagnosed health conditions. Among those with conditions, hypertension was most common (20%, n = 18), followed by diabetes (9%, n = 8) and asthma (7%, n = 6).

**Healthcare utilization:** Hospital visit frequency varied: annually (43%, n = 39), every six months (21%, n = 19), every three months (19%, n = 17), and monthly (18%, n = 16).

**Healthcare comfort levels:** Comfort visiting hospitals varied considerably: 44% (n = 40) felt comfortable only sometimes, 29% (n = 26) never felt comfortable, 18% (n = 16) felt comfortable, and 10% (n = 9) never visited due to discomfort. Mean comfort score was 2.3 on a 4-point scale (SD = 0.9), indicating generally low comfort levels possibly reflecting experiences of discrimination, stigma, or inadequate provider sensitivity.

### Substance use

Participants reported use of: tobacco (57%, n = 52), marijuana (7%, n = 6), alcohol (3%, n = 3), and heroin (2%, n = 2). Additional substances were reported by 21% (n = 19). The high tobacco prevalence may reflect coping mechanisms for social exclusion, economic stress, or limited access to mental health support.

### Mental Health Assessment (HSCL-25)

Mean HSCL-25 score was 2.1 (SD = 0.6, range: 1.2-3.8). Using the standard clinical cut-off of 1.75, 68% (n = 62) of participants exceeded the threshold for significant psychological distress, indicating probable anxiety and/or depressive disorder.

### Anxiety symptoms among the participants

The assessment of anxiety symptoms shows different levels of distress among participants (Table 2). For example, 28.6% did not experience feeling scared for no reason at all, while others felt it a little (45.1%), quite a bit (18.7%), or extremely (7.7%), with an average score of 2.05. Similar patterns were seen with fear, dizziness, nervousness, heart racing, trembling, tension, headaches, panic, and restlessness. Headaches were found to be most prevalent (mean = 2.57, SD = 0.89); 83% experienced at least “a little”**.** Restlessness reported with a mean = 2.20 (SD = 0.93)**. Similarly** Heart racing was reported mean = 2.11 (SD = 1.01), followed by Faintness/dizziness with a mean = 2.10 (SD = 0.95) and Tension mean = 2.08 (SD = 0.96)

**Table 2:** Mental Health Assessment of Khawaja Sira Participants via Hopkin Symptom Checklist (HSCL-25).

Statement	Not at all	A little	Quite a bit	Extremely	Mean
**Assessing Anxiety**					
Suddenly scared for no reason	26 (28.6%)	41 (45.1%)	17 (18.7%)	7 (7.7%)	2.05
Feeling fearful	29 (31.9%)	36 (39.6%)	21 (23.1%)	5 (5.5%)	2.02
Faintness, dizziness or weakness	26 (28.6%)	37 (40.7%)	21 (23.1%)	7 (7.7%)	2.10
Nervousness or shakiness inside	25 (27.5%)	41 (45.1%)	18 (19.8%)	7 (7.7%)	2.08
Heart pounding or racing	28 (30.8%)	35 (38.5%)	18 (19.8%)	10 (11%)	2.11
Trembling	32 (35.2%)	38 (42%)	19 (20.9%)	2 (2.2%)	1.90
Feeling tense or keyed up	26 (28.6%)	40 (44%)	17 (18.7%)	8 (8.8%)	2.08
Headaches	8 (8.8%)	39 (42.9%)	28 (30.8%)	16 (17.6%)	2.57
Spells of terror or panic	32 (35.2%)	37 (40.7%)	15 (16.5%)	7 (7.7%)	1.97
Feeling restless, can’t sit still	21 (23.1%)	40 (44%)	21 (23.1%)	9 (9.9%)	2.20
**Assessing Depression**					
Feeling low in energy	17 (18.7%)	39 (42.9%)	26 (28.6%)	9 (9.9%)	2.3
Blaming yourself for things	25 (27.5%)	31 (34.1%)	28 (30.8%)	7 (7.7%)	2.19
Crying easily	23 (25.3%)	35 (38.5%)	18 (19.8%)	15 (16.5%)	2.27
Loss of sexual interest or pleasure	31 (34.1%)	26 (28.6%)	23 (25.3%)	11 (12.1%)	2.15
Poor appetite	16 (17.6%)	41 (45.1%)	26 (28.5%)	8 (8.8%)	2.29
Difficulty falling asleep	17 (18.7%)	36 (39.6%)	26 (28.6%)	12 (13.2%)	2.36
Feeling hopeless about the future	18 (19.8%)	33 (36.3%)	24 (26.4%)	16 (17.6%)	2.42
Feeling blue	27 (29.7%)	33 (36.3%)	24 (26.4%)	16 (17.6%)	2.13
Feeling lonely	18 (19.8%)	38 (41.8%)	19 (20.9%)	16 (17.6%)	2.36
Thoughts of ending your life	37 (40.7%)	27 (29.7%)	16 (17.6%)	11 (12.1%)	2.01
Feeling of being trapped or caught	32 (35.2%)	30 (33.0%)	15 (16.5%)	14 (15.4%)	2.12
Worrying too much about things	11 (12.1%)	42 (46.2%)	24 (26.4%)	14 (15.4%)	2.45
Feeling no interest in things	17 (18.7%)	43 (47.3%)	18 (19.8%)	13 (14.3%)	2.30
Feeling everything is an effort	17 (18.7%)	42 (46.2%)	22 (24.2%)	10 (11.0%)	2.27
Feelings of worthlessness	22 (24.2%)	35 (38.5%)	17 (18.7%)	17 (18.7%)	2.32

### Depression symptoms among the participants

Based on the assessment, participants experienced various symptoms of depression. Many reported feeling low energy, with some experiencing extreme tiredness, while others felt only slightly tired or not at all. The data indicates that worrying excessively was the most common concern, with a mean score of 2.45 and a standard deviation of 0.91, and 88% reported experiencing at least some level of worry. The average score for suicidal ideation was 2.01 (SD = 1.0), and feelings of hopelessness had a mean of 2.42 (SD = 1.04). Sleep difficulties and loneliness were also prominent, with mean scores of 2.36 and standard deviations of 1.01 and 1.02, respectively. Additionally, feelings of worthlessness had a mean score of 0.9, and 27% of participants reported experiencing at least some thoughts of worthlessness. (Table 2)

**Gender differences:** Female-identified participants (n = 10) reported higher mean HSCL-25 scores (2.6, SD = 0.5) compared to intersex-identified participants (2.0, SD = 0.6), though small subgroup sizes limit interpretation.

### Health awareness

Table 3 presents data on health awareness among intersex individuals, highlighting their awareness and practices related to health monitoring and financial stability. Low health awareness was evident across multiple indicators. 41% (n = 37) of participants were aware of blood pressure, while 59% (n = 54) were unaware. Regarding blood sugar, 28% (n = 25) had knowledge, whereas 73% (n = 66) lacked awareness. Only 25% (n = 23) reported regular health check-ups, compared to 75% (n = 68) who did not. When it came to income sufficiency for basic needs, 33% (n = 30) said yes, while 67% (n = 60) said no. For disaster and emergency preparedness knowledge, 26% (n = 24) were informed, whereas 74% (n = 67) were not.These findings indicate substantial gaps in health literacy and preventive care engagement, compounded by financial constraints limiting access to routine health monitoring.

**Table 3:** Health awareness among Khawaja Sira individuals.

Statement	Yes N (%)	No N (%)	Mean
I know my blood pressure	37 (40.7%)	54 (59.3%)	1.59
I know my blood sugar	25 (27.5%)	66 (72.5%)	1.73
I have regular health check-ups	23 (25.3%)	68 (74.7%)	1.75
My income is enough for basic living expenses	30 (33.0%)	60 (65.9%)	1.67
I take the initiative to learn how to deal, with disasters and emergencies	24 (26.4%)	67 (73.6%)	1.74

### Health protective behaviours

Table 4 shows that many people do not follow healthy habits. Almost half rarely use vegetable oils instead of animal fats. The HPBS uses a 5-point response scale (1 = Never to 5 = Always), with higher scores indicating greater engagement in health-protective behaviours; a score of 3.0 represents the midpoint (“half a time”). Mean HPBS score was 2.5 (SD = 0.4, range: 1.6-3.8), indicating low to moderate engagement in health-protective practices, falling below the scale midpoint. Specific behaviours showed concerning patterns:. Specific behaviours showed concerning patterns:

**Table 4:** Health protective behavior among Khawaja Sira individuals.

Statement	Never	Rarely	Half a time	Frequent	Always	Mean
Use vegetable oils instead of animal oils in your daily routine	17 (18.7%)	45 (49.5%)	13 (14.3%)	13 (14.3%)	3 (3.3%)	2.34
Control the amount of salt used in your daily diet	15 (16.5%)	41 (45.1%)	12 (13.2%)	15 (16.5%)	8 (8.8%)	2.56
Limit the amount of sugar in your daily diet	18 (19.8%)	39 (42.9%)	14 (15.4%)	17 (18.7%)	3 (3.3%)	2.43
Eat half a pound to one pound of fresh vegetables every day	23 (25.3%)	41 (45.1%)	18 (19.8%)	8 (8.8%)	1 (1.1%)	2.15
Eat about half a catty of fruit every day	16 (17.6%)	39 (42.9%)	23 (25.3%)	10 (11.0%)	3 (3.3%)	2.40
I control my weight when I am over the normal weight	29 (31.9%)	29 (31.9%)	20 (22.0%)	9 (9.9%)	4 (4.4%)	2.23
I am worried about the current situation of food safety	23 (25.3%)	36 (39.6%)	16 (17.6%)	8 (8.8%)	8 (8.8%)	2.36
I engage in moderate-intensity activity for more than 30 minutes every day	25 (27.5%)	32 (35.2%)	20 (22.0%)	11 (12.1%)	3 (3.3%)	2.29
I persuade the smokers around me to quit smoking	37 (40.7%)	29 (31.9%)	11 (12.1%)	9 (9.9%)	5 (5.5%)	2.08
When someone is smoking around, I try to stay away	45 (49.5%)	26 (28.6%)	6 (6.6%)	8 (8.8%)	6 (6.6%)	1.95
I get enough sleep	7 (7.7%)	24 (26.4%)	34 (37.4%)	18 (19.8%)	8 (8.8%)	2.96
After I got sick, I followed the doctor’s medication instructions	9 (9.9%)	30 (33.0%)	18 (19.8%)	18 (19.8%)	16 (17.6%)	3.02
I throw away expired medicines	15 (16.5%)	28 (30.8%)	12 (13.2%)	10 (11.0%)	26 (28.6%)	3.04
I Understand the dangers of marriage between close relatives	12 (13.2%)	27 (29.7%)	15 (16.5%)	20 (22.0%)	17 (18.7%)	3.03
When driving or riding in a car, I use the seat belt correctly and in accordance with the regulations	19 (20.9%)	39 (42.9%)	15 (16.5%)	9 (9.9%)	9 (9.9%)	2.45
I use workplace protection measures	25 (27.5%)	26 (28.6%)	17 (18.7%)	11 (12.1%)	12 (13.2%)	2.55
When in strong sunlight, protect your skin by wearing appropriate clothing, using sunscreen skin care products or using a parasol	23 (25.3%)	29 (31.9%)	25 (27.5%)	9 (9.9%)	5 (5.5%)	2.38
I use water purification equipment (water purifier, purified water)	17 (18.7%)	30 (33.0%)	24 (26.4%)	13 (14.3%)	7 (7.7%)	2.59
I wear a mask in haze and windy weather	27 (29.7%)	36 (39.6%)	17 (18.7%)	8 (8.8%)	3 (3.3%)	2.16
I can adapt to new living, learning and working environment easily	15 (16.5%)	34 (37.4%)	21 (23.1%)	14 (15.4%)	7 (7.7%)	2.60
I can have fun in my free time	8 (8.8%)	31 (41.8%)	19 (20.9%)	17 (18.7%)	9 (9.9%)	2.79
I can relax and have fun by myself	12 (13.2%)	28 (30.8%)	24 (26.4%)	18 (19.8%)	9 (9.9%)	2.82
I get the help I need from others	10 (11.0%)	30 (33.0%)	30 (33.0%)	11 (12.1%)	10 (11.0%)	2.79
I am willing to take other people’s suggestions	7 (7.7%)	35 (38.5%)	26 (28.6%)	16 (17.6%)	7 (7.7%)	2.79
I will be nervous at some critical moments, but I can calm down quickly	9 (9.9%)	37 (40.7%)	26 (28.6%)	11 (12.1%)	8 (8.8%)	2.69
When I feel anxious, I change my mood by doing other things	12 (13.2%)	37 (40.7%)	22 (24.2%)	12 (13.2%)	8 (8.8%)	2.64
When I encounter difficulties, I find ways to solve it	9 (9.9%)	29 (31.9%)	23 (25.3%)	18 (19.8%)	12 (13.2%)	2.95

### Nutrition and dietary practices

Approximately 30% of individuals report frequently or always consuming 0.5 to 1 pound of vegetables daily, with a mean score of 2.15 and a standard deviation of 1.0. Similarly, only 14% regularly or always consume fruits daily, reflected by a mean of 2.40 and a SD of 1.0. Control over salt intake is maintained by 25% of the population, with a mean score of 2.56 and SD of 1.1, while 22% frequently or always control their sugar intake, indicated by a mean of 2.43 and SD of 1.0.

### Physical activity and sleep

Moderate exercise for more than 30 minutes daily was reported as occurring “frequently” or “always” by 15% of respondents, with a mean score of 2.29 and a standard deviation of 1.1. Adequate sleep was similarly reported by 29% of participants as “frequently” or “always,” with a mean score of 2.96 and a standard deviation of 1.1.

### Tobacco exposure

Approximately 15% of individuals frequently or always persuade others to quit smoking, with a mean score of 2.08 and a standard deviation of 1.2, while 41% never attempt to do so. Similarly, regarding avoidance of secondhand smoke, 15% do so frequently or always, with a mean of 1.95 and a standard deviation of 1.3, whereas half of the respondents never avoid it.

### Healthcare adherence

Following medication instructions, 37% of respondents report doing so “frequently” or ‘always,’ with a mean score of 3.02 and a standard deviation of 1.2. Regarding the disposal of expired medicines, 40% indicate they do this “frequently” or ‘always,’ with a mean of 3.04 and a standard deviation of 1.4.

Low engagement in health-protective behaviours may reflect multiple barriers including financial constraints (66% insufficient income), limited health literacy, and restricted access to resources rather than knowledge deficits alone.

### Bivariate analyses

Basic bivariate analyses were conducted to explore associations between key socioeconomic and health variables. Given the small, non-probabilistic sample, these analyses are intended to be hypothesis-generating rather than confirmatory, and all findings should be interpreted with caution.

**Income adequacy and regular health check-ups.** Among the 91 participants with complete data on both variables, participants who reported sufficient income for basic living expenses were significantly more likely to undergo regular health check-ups than those with insufficient income (40.0% vs. 18.3%). A Chi-square test with Yates’ continuity correction confirmed a statistically significant association (χ²(1) = 3.86, p = 0.049; OR = 2.97, 95% CI: 1.11–7.92).

**Employment status and psychological distress.** Participants without any paid employment (unemployed or student only; n = 46) were more likely to exceed the HSCL-25 clinical threshold for significant psychological distress (≥1.75) compared to those in any form of paid work (n = 45): 80.4% vs. 55.6%. This association was statistically significant (χ²(1) = 5.39, p = 0.020; OR = 0.30, 95% CI: 0.12–0.78), indicating that not being employed was associated with nearly 3.4 times higher odds of exceeding the distress threshold.

**HSCL-25 total scores by employment status.** HSCL-25 scores were significantly higher among participants without paid employment (Median = 2.21, IQR: 1.90–2.53) compared to those in paid work (Median = 1.97, IQR: 1.55–2.34), with a Mann-Whitney U test confirming the difference (U = 770.5, p = 0.036, r = 0.22, indicating a small-to-moderate effect size).

**HSCL-25 total scores by educational attainment.** Participants with no formal education (n = 46) reported significantly higher HSCL-25 scores (Median = 2.38, IQR: 1.90–2.75) than those with any formal schooling (n = 45; Median = 1.95, IQR: 1.56–2.30). This difference was statistically significant (U = 685.5, p = 0.006, r = 0.29, a moderate effect size). These findings are consistent with existing literature linking socioeconomic marginalisation, specifically unemployment and limited educational attainment—to poorer mental health outcomes in gender-diverse populations (Table 5).

**Table 5:** Summary of bivariate analyses (n = 91).

Analysis	Test	Statistic	p-value	Key Finding
Income adequacy × Regular health check-ups	Chi-square (Yates)	χ²(1) = 3.86	0.049	Sufficient income associated with more check-ups (OR = 2.97, 95% CI: 1.11–7.92)
Employment status × HSCL-25 distress (≥1.75)	Chi-square (Yates)	χ²(1) = 5.39	0.020	Not employed associated with higher distress odds (OR = 0.30 for employed, 95% CI: 0.12–0.78)
HSCL-25 scores ~ Employment status	Mann-Whitney U	U = 770.5	0.036	Not employed: Median 2.21 vs. Employed: Median 1.97 (r = 0.22)
HSCL-25 scores ~ Educational attainment	Mann-Whitney U	U = 685.5	0.006	No education: Median 2.38 vs. Any schooling: Median 1.95 (r = 0.29)

*All analyses are hypothesis-generating only. Small non-probabilistic sample limits inferential interpretation. p < 0.05 considered statistically significant.*

## Discussion

This study highlights the complex challenges faced by the Khawaja Sira community in Islamabad, emphasizing notable health disparities and socio-economic obstacles. Similar patterns are evident in international research, where gender-diverse individuals frequently encounter discrimination and have restricted access to healthcare services. For example, a study conducted in the United States indicates that participants are more prone to mental health issues, such as anxiety and depression, largely due to societal stigma [15]. The data further shows that the health issues confronting participants are diverse, encompassing mental health concerns and physical conditions like hypertension and diabetes. These issues are often worsened by societal stigma and inadequate healthcare access, underscoring the urgent need for comprehensive healthcare strategies tailored to their needs.

Socio-economic factors significantly influence the overall well-being of Khawaja Sira community members. The study indicates that a considerable portion of this community experiences a lack of family support and high unemployment rates, which directly hinder their ability to access essential healthcare services. Economic marginalization is further intensified by societal discrimination and stigmatization, making it even more challenging for participants to seek appropriate medical care and support. Many participants report feeling uncomfortable or unsafe when visiting healthcare facilities, underscoring the critical need for healthcare providers to undergo targeted sensitization and training. Such efforts are vital to foster a more inclusive, respectful, and supportive environment. These findings are consistent with research conducted in Australia, where gender-diverse individuals face similar barriers to healthcare access, highlighting the global nature of these challenges [16].

The study provides valuable insights into the behavioral and socio-economic challenges faced by the Khawaja Sira community. It highlights a high occurrence of tobacco use among participants, which appears to serve as a coping strategy against stress and discrimination they frequently encounter. This underscores the urgent need for specialized mental health and addiction support services tailored to their unique experiences.

Furthermore, the study reveals that educational attainment within the Khawaja Sira community is markedly low, with many individuals having never accessed formal schooling. This educational deficit hampers their employment prospects, thereby perpetuating cycles of poverty and social marginalization. To break this cycle, it is crucial to implement inclusive education policies and programs that promote access and equity for gender-diverse individuals.

This finding emphasises the diverse presentation of depression symptoms among participants. The range in symptom severity, from mild to severe, and the variety of experiences—from feelings of sadness and guilt to thoughts of self-harm—highlight the complexity of depression as a mental health issue. These results align with another study showing common diagnoses such as depression, anxiety, arthritis, and hypertension, with notable variations by age [15]. Nearly a third of participants reported difficulty with everyday tasks, and over half experienced significant challenges with cognitive functions. The higher reported rates of symptoms like worthlessness and worry suggests these could be key indicators within this group. Conversely, the lower occurrence of suicidal ideation and feelings of being trapped might reflect resilience or differences in symptom reporting. Overall, these findings underscore the need for personalised assessment and intervention strategies to address depression’s multifaceted nature. Additionally, research shows a direct link between financial stability and better health outcomes, supporting improvements in both economic and overall health [17].

Besides educational and mental health concerns, the study highlights that participants often face rejection, exclusion, and discrimination at work, which further hinders their socio-economic mobility. These findings align with other research that shows, similarly, gender-diverse individuals tend to have lower educational levels. They are more likely to experience rejection, exclusion, and discrimination at work, and face significant barriers in healthcare environments [18]. They also encounter notable obstacles within healthcare settings, restricting their access to essential medical services. Addressing these complex issues requires comprehensive interventions that foster acceptance, equality, and support across all areas of society.

Overall, the study emphasises the critical importance of implementing targeted interventions and comprehensive policy reforms to effectively address the multifaceted health and socio-economic challenges faced by the Khawaja Sira community in Islamabad. It advocates for the adoption of multifaceted strategies that not only raise health awareness but also significantly improve access to quality healthcare services and expand socio-economic opportunities. Such measures are crucial for promoting meaningful and sustainable enhancements in the overall quality of life for Khawaja Sira community members. Achieving this requires a concerted and collaborative effort among policymakers, healthcare providers, community organizations, and advocacy groups to build a more inclusive, understanding, and equitable society. On a global scale, similar recommendations have been made, underscoring the necessity of promoting the rights, health, and well-being of gender-diverse individuals worldwide, and recognizing that these issues are interconnected across different regions and cultural contexts [19].

### Study limitations

The study has a number of limitations. Firstly, sampling bias arises from snowball recruitment through Gurus, likely overrepresenting connected network members and excluding isolated or marginalized community segments. Our ethnic composition, with 74% Punjabi, probably reflects network clustering rather than the actual population distribution. Additionally, using census figures (n = 133) instead of CBO estimates (>13,000) for sample calculation likely compromised representativeness. The cross-sectional design prevents establishing causal relationships between socioeconomic factors and health outcomes. The small sample size limits subgroup analyses and statistical testing of associations. Sensitive topics such as substance use and suicidal ideation may be underreported due to social desirability bias, despite interviewer training and private settings. While instruments were translated, limited cultural adaptation may impact construct validity for this population. Lastly, the findings are specific to Islamabad’s sociocultural context and may not be generalizable to other regions of Pakistan or beyond.

### Implications for policy and practice

Healthcare system reforms include implementing mandatory cultural competency training for healthcare providers on gender-diverse care. Additionally, establishing dedicated gender-affirming health services with trained staff can improve patient outcomes. Integrating routine mental health screening alongside low-cost counseling services further supports community well-being. Developing community health worker programs that embed trusted Khawaja Sira community members ensures culturally sensitive care.

In terms of social protection, expanding income support programs targeting unemployed or underemployed Khawaja Sira individuals is crucial. Creating employment quota systems with effective enforcement mechanisms can promote inclusivity. Furthermore, establishing anti-discrimination complaint mechanisms supported by legal aid helps address grievances and protect rights.

Education and empowerment efforts should focus on implementing inclusive policies that guarantee access for gender-diverse youth. Developing adult literacy programs within Khawaja Sira community spaces can facilitate greater educational opportunities. Additionally, creating health literacy initiatives that emphasize preventive care and chronic disease management empowers individuals to take control of their health.

Research priorities involve conducting larger, probability-based studies with improved population estimates. Employing longitudinal designs enables the examination of health trajectories and the impact of interventions over time. Incorporating participatory research approaches that center community voices ensures relevance and cultural sensitivity. Lastly, investigating the intersectionality of gender identity with ethnicity, religion, and regional variation can provide a more comprehensive understanding of diverse experiences.

## Conclusion

This study documents substantial health inequities among Islamabad’s Khawaja Sira community, including high psychological distress, limited preventive care access, and socioeconomic marginalization. While the 2018 Protection of Rights Act represents legal progress, implementation gaps persist. Addressing these disparities requires multi-level interventions: healthcare system reforms promoting cultural competency, social protection policies reducing economic vulnerability, and structural changes dismantling discrimination. Future research should employ more representative sampling, longitudinal designs, and participatory approaches to better understand and address the complex determinants of health inequity among Pakistan’s gender-diverse populations.
